# Predicting congenital renal tract malformation genes using machine learning

**DOI:** 10.1038/s41598-023-38110-z

**Published:** 2023-08-14

**Authors:** Mitra Kabir, Helen M. Stuart, Filipa M. Lopes, Elisavet Fotiou, Bernard Keavney, Andrew J. Doig, Adrian S. Woolf, Kathryn E. Hentges

**Affiliations:** 1 CentreDivision of Evolution, Infection and Genomics, Faculty of Biology, Medicine and Health, Manchester Academic Health Science Centre, The University of Manchester, Oxford Road, Manchester, M13 9PT UK; 2 Manchester Centre for Genomic Medicine, St. Mary’s Hospital, Health Innovation Manchester, Manchester University Foundation NHS Trust, Manchester, M13 9WL UK; 3 Division of Cell Matrix Biology and Regenerative Medicine, School of Biological Sciences, Faculty of Biology, Medicine and Health, The University of Manchester, Manchester, M13 9PL UK; 4 Division of Cardiovascular Sciences, School of Medical Sciences, Faculty of Biology, Medicine, and Health, The University of Manchester, Manchester, M13 9PL UK; 5 Manchester Heart Institute, Manchester University NHS Foundation Trust, Manchester Academic Health Science Centre, Manchester, M13 9WL UK; 6 Division of Neuroscience, School of Biological Sciences, Faculty of Biology, Medicine and Health, University of Manchester, Stopford Building, Manchester, M13 9BL UK; 7 Department of Nephrology, Royal Manchester Children’s Hospital, Manchester Academic Health Science Centre, Manchester, M13 9WL UK; 8 Present Address: C.B.B Lifeline Biotech Ltd, 5 Propontidos Street, Strovolos, 2033 Nicosia, Cyprus

**Keywords:** Computational biology and bioinformatics, Developmental biology, Genetics, Diseases

## Abstract

Congenital renal tract malformations (RTMs) are the major cause of severe kidney failure in children. Studies to date have identified defined genetic causes for only a minority of human RTMs. While some RTMs may be caused by poorly defined environmental perturbations affecting organogenesis, it is likely that numerous causative genetic variants have yet to be identified. Unfortunately, the speed of discovering further genetic causes for RTMs is limited by challenges in prioritising candidate genes harbouring sequence variants. Here, we exploited the computer-based artificial intelligence methodology of supervised machine learning to identify genes with a high probability of being involved in renal development. These genes, when mutated, are promising candidates for causing RTMs. With this methodology, the machine learning classifier determines which attributes are common to renal development genes and identifies genes possessing these attributes. Here we report the validation of an RTM gene classifier and provide predictions of the RTM association status for all protein-coding genes in the mouse genome. Overall, our predictions, whilst not definitive, can inform the prioritisation of genes when evaluating patient sequence data for genetic diagnosis. This knowledge of renal developmental genes will accelerate the processes of reaching a genetic diagnosis for patients born with RTMs.

## Introduction

The mammalian renal tract (RT) comprises the kidneys and lower urinary tract, with the latter containing the ureter, bladder and urethra. Congenital RT malformations (RTMs), also known as congenital anomalies of the kidney and urinary tract (CAKUT), are the major cause of severe kidney failure in young children, and are found in around 20% of young adults with this condition^1,2^. In the last three decades^3,4^, monogenic causes have been discovered for human RTMs. Nevertheless, these studies have identified defined genetic causes for only a minority of human RTMs, with hepatocyte nuclear factor 1B (*HNF1B)* and paired box-2 (*PAX2)* being two of the commoner genes implicated^5,6^. While poorly defined environmental perturbations may cause RTMs in some cases by disrupting renal organogenesis^7,8^, it is very likely that pathological variations in additional genes have yet to be identified. Progress in discovering further genetic causes for RTMs are, in part, limited by challenges in prioritising candidate genes from RTM patient sequence analysis, or within genomic regions implicated by association or linkage studies.

Machine learning algorithms can classify genes into groups based on sequence and network properties^9,10^. These properties are termed ‘features’. Supervised machine learning classifiers then utilise two training datasets of these features: one training set contains examples (in this case, genes) known to be associated with the condition or property sought, and the other set includes examples known to not possess that condition or property. A machine learning classifier is then generated by using the properties of the two training sets in an optimal way to separate the groups. Once trained, the classifier can be applied to predict the correct group for a new example.

We have previously used machine learning to identify proteins that constitute drug targets^11–13^ and to identify genes essential for mammalian embryonic development^14^. Others have implemented machine learning to identify genes that drive kidney clear cell cancer^15^ and to assign roles of genetic variants to kidney excretory function^16^. Here we exploit supervised machine learning to identify genes with a high probability of being involved in renal development. These genes, when mutated, would therefore be promising candidates for causing RTMs. Due to the limited knowledge of genetic causes of human RTMs, we developed a positive training set of genes known to cause RTMs when mutated in the mouse, and a second training set of genes known not to cause disruptions to renal tract development. We utilised the mouse as a model organism because it is heavily studied, and mouse knockout experiments have proved useful in revealing biological functions of many human genes^17–19^. By applying supervised machine learning to the features of the genes in these two training sets, the classifier determines which feature values are common to renal developmental genes, and then identifies genes possessing these attributes from a novel dataset. Here we report the kidney development association status for all genes in the mouse genome as predicted by our classifier. Due to developmental similarities and genetic conservation between mouse and human, the genes we predict to have a role in mouse RTM development will comprise a dataset worthy of further investigation for human genetic diagnosis. Overall, our predictions can inform the prioritisation of candidate genes and accelerate the processes of reaching a genetic diagnosis for individuals affected by RTMs.

## Results

### Datasets

**Figure 1:**
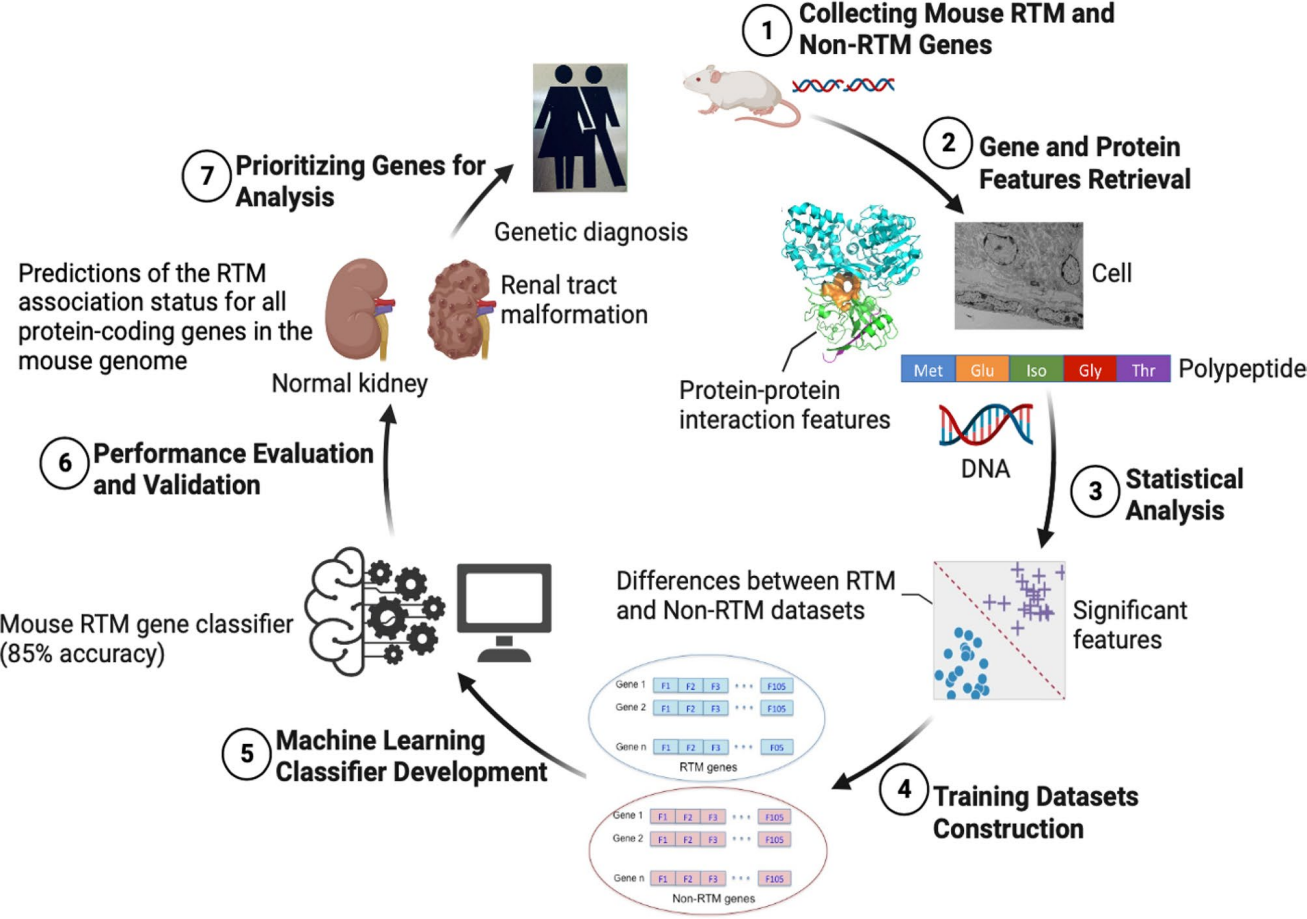
The workflow for predicting mouse RTM genes integrating genomic and protein features using Random Forest classification model. First, features of mouse genes are collated from public databases. Statistical analyses and feature selection were then performed to identify most informative features differentiating between known RTM and non-RTM genes. A Random Forest classifier was built to predict RTM and non-RTM genes from these features. Finally, this classifier was used to predict RTM association status for all protein coding genes in the mouse genome not included in the classifier development.

We first compiled a dataset of genes that are known to cause RTMs when mutated in the mouse, and a dataset of genes that are known not to cause RTMs (non-RTM) (Fig. 1), using data from the Mouse Genome Informatics (MGI)^20^ database and data from the IMPC consortium^21^. This gave 310 mouse genes that are associated with RTMs when mutated (hereafter called ‘RTM genes’) and 4752 genes known not to cause documented RTM developmental defects (‘non-RTM genes’), based on phenotype annotations of null alleles of targeted single-gene knockouts. RTM genes were also manually verified for their roles in human RT development based on the literature and RTM disease associations^22^. Human CAKUT-causing genes^23^ are included in our training set. In order to investigate features specific to protein function, we restricted our datasets to protein-coding genes only. As a result, we obtained a total of 174 RTM and 4141 non-RTM mouse genes (Tables S1 and S2).

### Properties of RTM and non-RTM genes

We collected data for a wide range of genomic and proteomic features of mouse protein coding genes^24^, including gene and protein length, gene expression, subcellular localisation, and known interaction partners. A total of 106 features of mouse genes linked to RT development were compared to genes not associated with RTM to reveal properties linked to RT development. Many features were found to be statistically significantly different in their distributions between the RTM and non-RTM datasets (Table 1).

**Table 1:** List of statistically significant features between RTM and non-RTM genes. The median value of each feature is reported. Statistically significant results are listed for *P*–values less than 0.05.

Features	RTM	Non-RTM	*P*-value
Gene Length (bp)	29,459	24,666	1.3 × 10^–2^
Exon length (bp)	3672	2940	4.5 × 10^–5^
Intron length (bp)	25,433.5	21,268	2.7 × 10^–2^
Molecular weight (Da)	54,489.96	51,952.64	3.1 × 10^–2^
Protein length (aa)	497.5	465	2.0 × 10^–2^
Aliphatic (%)	26.67	27.64	1.3 × 10^–3^
Glu (%)	5.74	6.35	7.8 × 10^–4^
Gly (%)	7.11	6.44	3.8 × 10^–4^
Ile (%)	3.99	4.26	2.1 × 10^–3^
Leu (%)	9.17	9.85	9.7 × 10^–4^
Asn (%)	3.79	3.53	1.6 × 10^–2^
Pro (%)	6.27	5.67	4.1 × 10^–3^
Gene expression across eight-week fibroblast tissues	5.61	2.18	1.6 × 10^–3^
Gene expression across post-juvenile RTM tissues (female) (FPKM)	11.07	3.65	5.3 × 10^–7^
Gene expression across post-juvenile RTM tissues (mixed) (FPKM)	6.89	2.84	5.0 × 10^–6^
Gene expression across post-juvenile RTM tissues (male) (FPKM)	6.56	2.12	1.0 × 10^–5^

**Figure 2:**
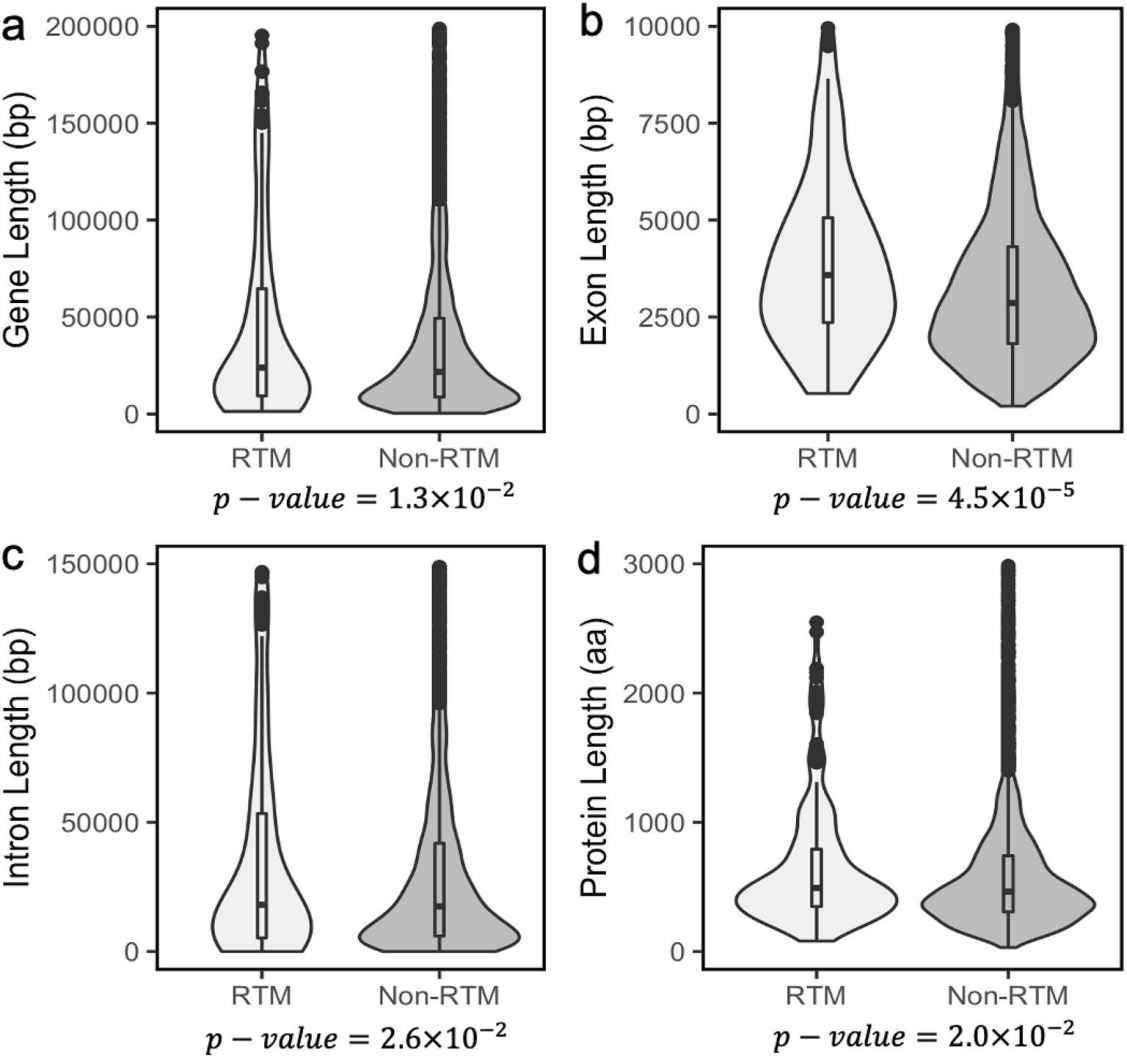
Distributions of the total gene length, exon length, intron length and protein length in RTM and non-RTM datasets. These violin plots outline distribution of (**a**) gene length (**b**) exon length (**c**) intron length and (**d**) protein length with overlaid boxplots. The width of the violin plots represents the proportion of the data located there; the top and bottom of the boxplots denote the upper and lower quartiles; the line inside the box denotes the median of the data. The *P-*values from the Mann–Whitney U tests are reported below their respective graphs.

We found that RTM genes are more likely to be longer in length than non-RTM genes (Table 1, Fig. 2a). Additionally, RTM genes tend to have both longer exons and longer introns than non-RTM genes (Table 1, Fig. 2b,c). A greater proportion of RTM genes are expressed at the organogenesis stage of mouse development when compared with non-RTM genes (74.7 vs. 57.8%, Chi-squared *P-*value: 4.1 × 10^–3^). RTM genes were also highly expressed in eight-week fibroblast and post-juvenile RT tissues (Table 1).

**Figure 3:**
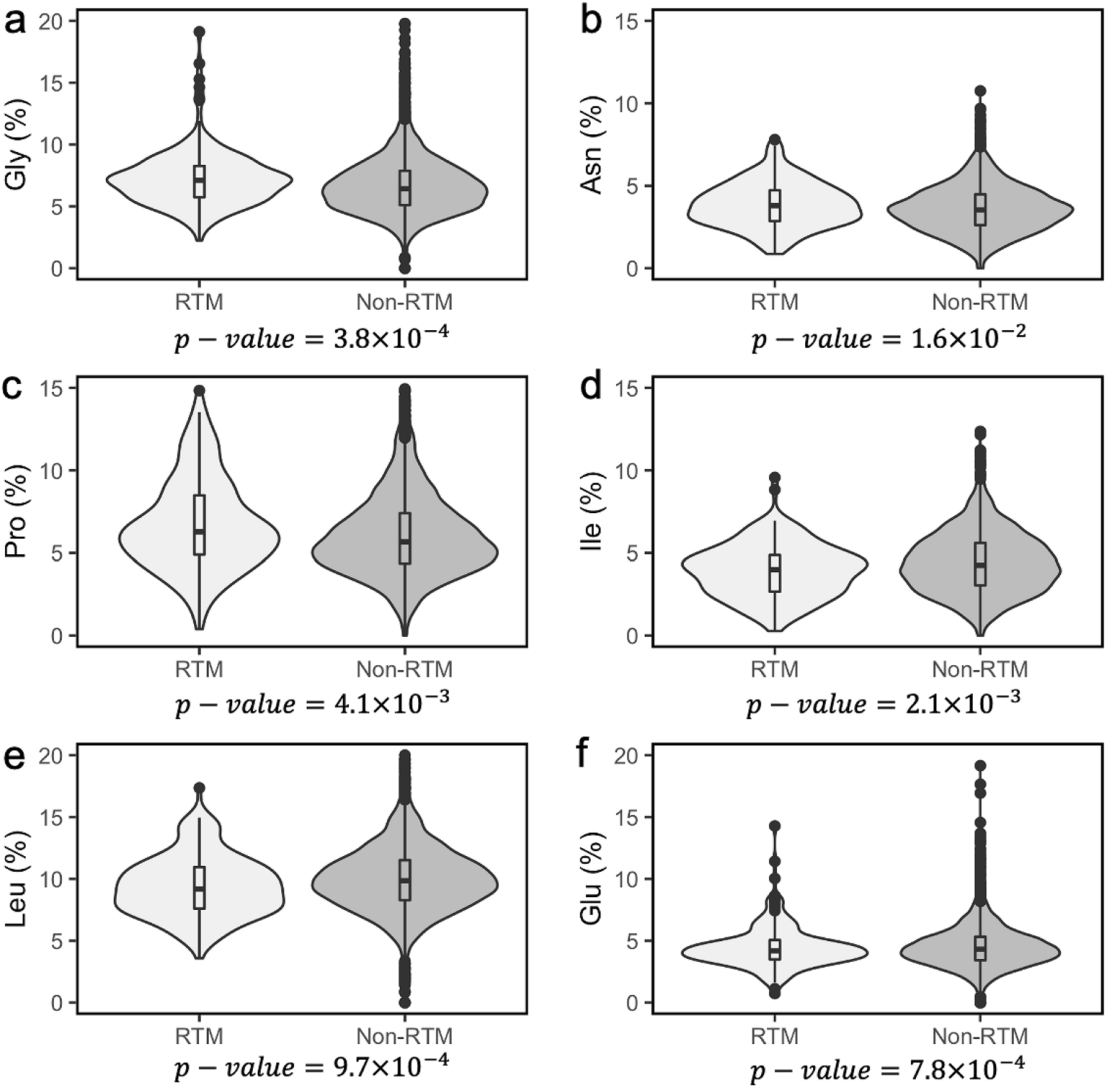
Distributions of several amino acid residues (%) between RTM and non-RTM mouse proteins. These violin plots outline distribution of the proportion of (**a**) glycine (**b**) asparagine (**c**) proline and (**d**) isoleucine (**e**) leucine (**f**) glutamine residues with overlaid boxplots. The width of the violin plots represents the proportion of the data located there; the top and bottom of the boxplots denote the upper and lower quartiles; the line inside the box denotes the median of the data. The *P*-values from the Mann–Whitney U tests are reported below their respective graphs.

Proteins encoded by RTM genes are likely to be longer (Table 1, Fig. 2d) and have higher molecular weight than proteins encoded by non-RTM genes (Table 1). RTM proteins have Glycine (Gly), Asparagine (Asn) and Proline (Pro) residues in greater proportions (Fig. 3a–c). In contrast, non-RTM proteins have more Isoleucine (Ile), Leucine (Leu), and Glutamine (Gln) residues (Fig. 3d–f). Non-RTM proteins are also likely to have more aliphatic residues (Table 1). We found that the RTM dataset has more N-linked glycoproteins than the non-RTM dataset (48.3 vs. 34.7%, Chi-squared *P-*value = 3.0 × 10^–3^) and has a higher proportion of proteins that contain signal peptides (40.2 vs. 27.4%, Chi-squared *P-*value = 1.6 × 10^–3^). Based on UniProt^25,26^ keyword annotation, RTM proteins are more likely to be associated with regulating the transcription of genes (21.7 vs. 13.3%, Chi-squared *P*-value = 5.2 × 10^–3^). We obtained annotations of six primary enzyme classes from UniProt and counted the number of RTM and non-RTM proteins belonging to these classes. The non-RTM dataset is enriched for hydrolase enzymes as compared with its RTM counterpart (5.2 vs. 10.7%, Chi-squared *P*-value = 2.6 × 10^–2^). Signal peptide motifs are more frequent in proteins encoded by RTM genes compared to proteins encoded by non-RTM genes (40.2 vs. 27.7%, Chi-squared *P*-value = 1.6 × 10^–3^).

Gene Ontology (GO)^27^ is one of the most widely used approaches for annotating gene functions. We found differences in the GO term annotations for the biological process and cellular component classes between RTM and non-RTM gene groups. For biological processes, GO terms enriched in the RTM dataset include ‘kidney development’, ‘uretic bud morphogenesis’, ‘uretic bud development’, ‘metanephros development’, and ‘mesonephros development’. Terms enriched in the non-RTM dataset include ‘inflammatory response, ‘immune system process’, ‘apoptotic process’, and ‘ion transport’. For cellular component, terms most frequently associated with RTM genes include: ‘extracellular region’, ‘basement membrane’, ‘cell surface’ and ‘extracellular matrix’. Non-RTM dataset was enriched for terms including ‘glutamatergic synapse’, ‘membrane’, ‘cytoplasm’, ‘plasma membrane’, and ‘cytosol’. Lists of the 20 most enriched GO terms for each class are listed in Tables S3–S6.

Known protein–protein interaction (PPI) data for mouse proteins were also analysed. This PPI network contains all known literature-curated interactions of mouse proteins from BioGrid^28^, BIND^29^, Chen PiwiScreen^30^, IntAct^31^, INNATEDB^32^, MGI, DIP^33^, MINT^34^ and also from a recent study^35^. We found three statistically significant properties in the PPI network: Betweenness centrality and bottleneck of RTM proteins in the interaction network is significantly higher than that of non-RTM proteins (*P*-value = 2.8 × 10^–4^ and *P*-value = 4.8 × 10^–2^, respectively). In contrast, the eigenvector score which measures the centrality of a protein in the interaction network is significantly higher for non-RTM proteins than RTM-proteins (*P*-value = 7.2 × 10^–3^).

### Training and test datasets

Numerous features are significantly different between RTM and non-RTM genes. We therefore sought to develop a machine learning classifier that could categorise a mouse gene as RTM or non-RTM from its features (Fig. 1). We used 106 features as input to generate training datasets for classification. Our original dataset containing 174 RTM and 4141 non-RTM mouse genes had a severely imbalanced class frequency ratio (1:23.8). Imbalanced training datasets pose problems for machine learning strategies^36,37^; therefore, class distribution was balanced by oversampling the genes of the RTM (minority) class when training the classifiers. We generated balanced training datasets having 522 genes each from the RTM and non-RTM datasets. The 522 non-RTM genes were randomly selected from the 4141 non-RTM genes. The RTM dataset which had 174 genes was increased by an additional 348 genes, synthesized from the existing RTM genes. We applied the Synthetic Minority Oversampling Technique (SMOTE)^38^ to generate these synthetic RTM genes. These genes were close in feature space to the existing RTM genes. We then trained our classifier with this class-balanced dataset.

To evaluate the performance of our machine learning classifier, we assembled test datasets with genes that were not included in the training datasets (Fig. 1). Test 1 dataset (Table S7) contains 3619 genes from our original non-RTM dataset that were not used in classifier training. Test 2 dataset (Table S8) includes 27 mouse genes that are orthologues of those in the critical region involved in DiGeorge Syndrome. This chromosomal disorder occurs due to the deletion of a number of genes on chromosome 22q11.2, and the functions of many of these genes are still unknown. We utilised 22q11.2 deletion region genes as a test dataset because approximately 30% of DiGeorge patients have congenital kidney and/or and urinary tract anomalies^39,40^. Test 3 dataset (Table S9) includes 31 mouse orthologues of human genes from the non-syndromic vesicoureteric reflux (VUR) candidate region on human chromosome 10q26^41^; this region showed strong association with ureter malformation. Test 4 dataset (Table S10) comprises a total of 13,379 mouse protein-coding genes that have no experimental annotations for renal anomalies. The MouseMine^42^ database was used to retrieve these genes. Gene and protein features were then collected for the test dataset genes following the same procedure used for the training genes.

### Performance of the machine learning classifier

We performed feature selection prior to the training procedure. Feature selection is a useful tool for developing a classifier from a dataset with many features. It selects the most useful features from the training dataset and helps the classifier to learn a more efficient way to make predictions. Here, the Information Gain feature selection method in Weka has been used to identify the most important mouse gene features for classification from the training dataset. This method found a subset of 71 informative features amongst the 106 total features (Table 2 and S11). Most of these selected features were found to be statistically different in values between the RTM and non-RTM genes in this study, confirming their value as discriminators between the training sets.

**Table 2:** Top 10 features selected from the training dataset using the Information Gain feature selection method. Features are sorted in descending order with respect to the corresponding information gain value, with the most informative feature listed first.

Information gain	Feature	Feature type	Observation for RTM genes
0.216	Betweenness centrality	PPI network	High
0.202	Density of maximum neighborhood component (DMNC)	PPI network	Low (not statistically significant)
0.193	Bottleneck	PPI network	High (not statistically significant)
0.193	Organogenesis	Developmental stage	Expressed in high proportion
0.192	Glycoprotein	Post-translational modification	High proportion of N-linked glycosylated RTM proteins
0.191	Maximum neighborhood component (MNC)	PPI network	High
0.181	Cytoplasm UniProt	Subcellular location	Low proportion of cytoplasmic RTM proteins
0.178	Signal peptide	Protein sequence	More frequent
0.174	Nucleus uniprot	Subcellular location	Likely to be located in nucleus
0.171	Blastocyst	Developmental stage	Expressed in high proportion (not statistically significant)

To construct our machine learning classifier we used the Random Forest^43^ implementation in Weka^44^ which is an ensemble classifier comprising multiple decision tree models. It has been found to be a highly accurate machine learning method in numerous studies^14,45–47^. We employed tenfold cross-validation to increase the robustness of our classifier and mitigate the potential for classifier overfitting^48^. A classifier overfits if its prediction accuracy is higher on the training dataset than on the validation/test dataset. We observed that the cross-validation accuracy of our Random Forest classifier built on 70 selected features is 85.3% (891/1044) with 424 true-positives (TPs) (RTM genes correctly identified as RTM), 98 false-negatives (FNs) (RTM genes identified as non-RTM), 472 true-negatives (TNs) (non-RTM genes identified as non-RTM) and 50 false-positives (FPs) (non-RTM genes identified as RTM). Table 3 demonstrates the robust performance of this classifier by means of several performance metrics. We also compared the performance of this Random forest classifier with the J48 decision tree^49^, Gradient Boosted Tree (XGBoost)^50,51^ and Support Vector Machine (SVM)^52^ models. J48 classifier was developed in Weka, and XGBoost and SVM classifiers were implemented in R with default parameters settings using the tenfold cross-validation method. Table 3 shows the superiority of the Random Forest classifier in predicting RTM genes among all classifiers.

**Table 3:** Tenfold cross validation performance of the Random Forest, SVM, XGBoost and J48 classifiers trained and evaluated on the training dataset. Data from before and after feature selection are presented. Here, TP = True Positive; FP = False Positive; ROC = Receiver Operating Curve; PRC = Precision-Recall Curve.

Classifiers	Accuracy (%)	Gene class	TP rate	FP Rate	Precision	F-Measure	ROC area (AUC)	PRC area
Random Forest	85.34	RTM	0.812	0.105	0.885	0.847	0.923	0.937
		Non-RTM	0.895	0.188	0.827	0.859	0.923	0.906
SVM	78.73	RTM	0.795	0.220	0.783	0.789	0.856	0.848
		Non-RTM	0.780	0.205	0.792	0.786	0.856	0.851
XGBoost	73.94	RTM	0.747	0.268	0.736	0.741	0.804	0.829
		Non-RTM	0.732	0.253	0.743	0.737	0.804	0.756
J48	75.48	RTM	0.772	0.262	0.746	0.759	0.771	0.740
		Non-RTM	0.738	0.228	0.764	0.750	0.771	0.709

Our classifier showed an accuracy of 84.3% on the Test 1 dataset, which only contains non-RTM genes. We further used this classifier to identify the status of mouse genes in the Test 2 dataset, each of which could be an orthologue of a possible candidate for causing the renal defects associated with DiGeorge Syndrome. Among all genes in this dataset, DiGeorge critical region 14 (*Dgcr14*)*,* zinc finger DHHC-type palmitoyltransferase 8 (*Zdhhc8*), CRK like proto-oncogene adaptor protein (*Crkl*), guanine nucleotide-binding subunit beta-like protein (*Gnb1l*), KLF transcription factor 8 (*Klf8*), and DiGeorge critical region 8 (*Dgcr2*) were predicted as RTM genes. The remaining genes were identified as non-RTM. Moreover, this classifier identified Transforming acidic coiled-coil containing protein 2 (*Tacc2*)*,* carboxypeptidase X and M14 family member 2 (*Cpxm2*) genes as RTM genes from the Test 3 dataset which contains mouse orthologues of the VUR candidate region on human chromosome 10q26.

To test whether the Random Forest classifier suffers from overfitting, we generated 9 more balanced training datasets containing different subsets of non-RTM genes. Nine different Random Forest classifiers were trained on these datasets (Table S12). We found that the mean accuracy of these classifiers is 85.9% with a standard deviation (SD) of 0.7%. This low SD indicates that all these classifier’s prediction performances are very similar. This result confirms that our classifier is not biased by the choice of genes in the training dataset, because if the subset of genes chosen for the training dataset impacted highly upon the classifier accuracy, a high SD would have been detected when multiple classifiers were compared.

### Prediction of all genes in the mouse genome

We created a fourth test dataset (Test 4) that contains all those protein-coding genes in the mouse genome that were not included in the RTM and non-RTM datasets. From this test dataset, our classifier predicted 19% (2534/13,379) of genes as RTM genes, and the remaining 81% (10,845/13,379) as non-RTM genes. We generated a ranked list of these RTM genes with their likelihood of being associated with RT development (Table S13). The top 10 predicted RTM genes are listed in Table 4. Three genes from our most highly confident predictions, *Scube3*^53^, *Sema3c*^54,55^, and *Rspo3*^56,57^, have been independently experimentally validated as causing renal developmental defects.

**Table 4:** Top 10 mouse RT genes predicted using our Random Forest classifier. The probability score (Confidence Score) output by our classifier (normalised in the 0–1 range) indicates the confidence level of a prediction result and tells the likelihood of a mouse gene in the test dataset being associated with RT development. The Confidence Score reports the fraction of decision trees in the Random Forest that predict the gene to be associated with RTMs. A score of 1 would reflect that 100% of decision trees classify that gene as RTM associated, corresponding to the strongest possible confidence in the prediction.

Gene name	Encoded protein and function	Confidence score
*Scube3*	Signal peptide, CUB and EGF-like domain-containing protein 3	0.885
*Prss23*	Serine protease 23	0.880
*Sema3c*	Semaphorin-3C	0.870
*Wnt5b*	Protein Wnt-5b	0.865
*Tfpi*	Tissue factor pathway inhibitor	0.860
*Rspo3*	R-spondin-3	0.850
*Atrn*	Attractin	0.850
*Ptpru*	Protein tyrosine phosphatase receptor type U	0.850
*Dcbld1*	Discoidin, CUB and LCCL domain-containing protein 1	0.848
*Angptl1*	Angiopoietin-related protein 1	0.845

### RTM gene database

To provide data on predicted RTM genes, a publicly available database named MoRTalGene (http://130.88.96.183/) has been created. This database shows the RT/non-RT status for all mouse genes, either from published literature or from our predictions. The confidence scores stating the predicted probabilities of the genes to be RTM can also be obtained from this database. A known or predicted mouse gene can be searched by multiple identifiers such as: gene name, MGI ID, Ensembl ID and UniProt ID. Lists of all RTM and non-RTM genes (both known and predicted) within the mouse genome or within a particular chromosome and/or genomic region can also be retrieved from the database. All search results can be downloaded as CSV files.

## Discussion

We aimed to facilitate the identification of RTM candidate genes by identifying genes in the mammalian genome that are associated with RT development. This knowledge may accelerate this process of achieving a genetic diagnosis for patients with congenital RTMs, because genes associated with renal tract development are likely to cause congenital RTMs when deleterious variants are present. Using supervised machine learning, we generated a Random Forest classifier that achieved 85% accuracy in tenfold cross validation trials after feature selection. Additionally, the classifier was 85% accurate when predicting the RTM association status of genes within our Test 1 dataset, which included all known non-RTM genes not used for training the classifier. We examined two genomic regions associated with RTMs, the 22q11.2 deletion region (DiGeorge Syndrome Critical Region) and the non-syndromic vesicoureteric reflux (VUR) candidate region on human chromosome 10q26. Here we present several candidate genes that can be examined through future experimental analysis as likely causative genes for the RTMs associated with these loci. Our database of RTM association status for all protein coding genes will be of value to researchers and clinicians investigating genetic causes of RTMs.

Our study identified properties of genes required for RT development. Although some of the properties are not surprising, such as high expression levels in the developing RT, others are more difficult to interpret, such as amino acid content. Some of the features more highly represented in RTM genes have also been found to be associated with genes required during mammalian development, such as longer sequence length, high betweenness centrality in the PPI network, high PPI network bottleneck score, and nuclear localisation, and therefore their inclusion in the RTM gene class is likely reflective of a developmental function for RTM genes.

Our genome predictions demonstrate that approximately 18% of protein-coding genes in the mouse genome may have a role in RT development, while 82% do not have such a role. These proportions are dissimilar to those of our initial training datasets compiled from the published literature, where we found that 174 protein-coding genes have been shown to be involved in RT development, compared to 4141 genes have been shown to not cause detectable RT phenotypes when mutated. However, it should be noted that some of the non-RTM genes may have had limited RTM characterisation, and therefore may in future with additional phenotyping be found to be RTM genes. Our classifier is not simply recapitulating the input proportions. The higher proportion of genes predicted to have a role in RT development as compared to those known to have a role in RTM development from experimental investigation indicates that RTM genes have been under-sampled in experimental studies. We therefore propose that further experimental analysis of the genes we predict as highly likely to be associated with RT development will reveal new gene functions and promising new models for congenital RTMs.

Our most highly confident predictions of RTM genes include several genes with links to renal disorders. For example, the *Scube3* gene is expressed during kidney development^58^. A *Scube3* mutant mouse harbouring a missense variant, *Scube3*^*N294K/N294K*^, has been identified from a mutagenesis screen^53^. These mice display alterations in renal function, including increased electrolyte, total protein, albumin, and glucose excretion rates. It has also been recently reported that bi-allelic inactivating variants in *SCUBE3* are associated with a skeletal and craniofacial developmental disorder linked to impaired BMP signalling^59^. It is unclear if kidney function was evaluated in these patients. However, BMP4 mutations cause defects in kidney development^60^, providing support for the hypothesis that altered SCUBE3 function can cause renal tract abnormalities due to the loss of BMP developmental signals. Additionally, *SCUBE3* has been identified as a renal cell carcinoma^61^ tumour suppressor gene. Erroneous hypermethylation of the promoter of *SCUBE3* in renal cell carcinoma leads to a 45% reduction in the expression level of the gene as compared to control kidney cell expression levels. Tumour methylation of *SCUBE3* also was associated with a significantly increased risk of death and cancer relapse. Together, these studies support our finding that *Scube3* is a gene of relevance to RT development.

Bioinformatic analysis of renal cell carcinoma transcriptome datasets has revealed that *PRSS23* displays significant differential expression between tumour and non-tumour datasets^62^. Further support for a role for *PRSS23* in kidney function comes from transcriptome studies of patients with focal segment glomerulosclerosis (FSGS), which is a major cause of end stage renal disease. FSGS patients exhibit upregulation of *PRSS23*, as does the *Cd2ap*^+*/-*^*, Fyn*^*-/-*^ mouse model of FSGS^63^. It is hypothesised that PRSS23 may promote TGFB signalling and cause renal tissue damage^63^. Whether interactions between PRSS23 and TGFB occur during kidney development remains a question for further investigation.

A role for *Sema3c* kidney development has been noted in a mutant mouse model, whereby *Sema3c* mutants showed reduced ureteric bud branching^55^. This mouse model incorporated the use of a GFP reporter, and therefore was not included in our training set genes which are exclusively targeted deletion models^55^. Furthermore, a recent study reports that the *Sema3c* gene is associated with the pathophysiology of acute kidney injury^54^. *Sema3c* knockout mice display decreased renal tissue damage and leukocyte infiltration following acute kidney injury. *Sema3c* is expressed in the wild type developing mouse kidney, but this expression is no longer detectable in the adult^54,64^. However, after surgically induced acute kidney injury *Sema3c* expression is upregulated as compared to control uninjured kidneys. Analysis of kidney biopsies from patients with acute injury also confirms upregulation of *SEMA3C*, indicating conservation of its function. Secretion of Sema3C protein following injury was detected, leading to the hypothesis that damaged kidney tubules produce Sema3c which causes further renal vascular damage and reduced blood flow.

In a study to identify genes driving early events in the formation of Wilms tumours, or nephroblastomas, the gene *WNT5b* was identified to have upregulated expression in human Wilms tumour blastemal cells as compared to differentiated kidney glomerular cells^65^. WNT5B protein expression was detected in human developing kidneys subsequent to renal vesicle formation, with expression in the nuclei of differentiated kidneys and in the cytoplasm in Wilms tumour tissue. Wilms tumours also often display an increase in copy number of *WNT5B*^66^, suggesting this gene may be involved in tumour pathogenesis. These studies indicate that disruption of Wnt signalling, and in particular increased *WNT5B* expression, may disrupt nephrogenesis.

*Tfpi* encodes a secreted protease inhibitor produced by kidney myofibroblasts, which likely has a role in the pathology of autosomal dominant polycystic kidney disease^67^. Myofibroblast depletion reduces kidney cyst growth and cyst epithelial cell proliferation in an autosomal dominant cystic kidney disease mouse model. It is hypothesised that the secretion of protease inhibitors, such as Tfpi, by myofibroblasts promotes the proliferation of cyst epithelium, leading to worsening renal function and advanced disease progression.

The R-spondin genes *Rspo1* and *Rspo3* are expressed in the developing mouse kidney from embryonic day (E) 10.5, in an overlapping pattern with *Six2*+ renal progenitors^57^. By late gestation, *Rspo3* is strongly expressed in the cortical stroma compartment and stroma cells lining ducts of the renal papilla. Kidney-specific deletion of *Rspo3* results in a mild reduction of renal progenitor cells, whereas joint deletion of *Rspo1* and *Rspo3* resulted in severe renal hypoplasia. Further characterisation of *Rspo3* in the mouse developing kidney stroma revealed a requirement for *Rspo3* in the stromal compartment to maintain kidney progenitor cells in late gestation. Additionally, single cell transcriptomics studies have identified *Rspo3* as a key marker of the kidney stromal compartment^56^. Further investigation of these genes in human renal tract malformations and congenital disease is needed. The *Rspo3* knockout experimental studies were performed after the compilation of our RTM training set, and thus the abnormal renal developmental phenotype of the mouse knockout model was not yet known when our computational study was initiated.

Another gene within our top 20 most confident predictions of genes associated with RT development is the gene *Slit3*. At the start of our study this gene was not annotated in MGI as being associated with RTMs using the molecular phenotyping terms we selected for inclusion as an RTM gene. However, a recent report confirms that *SLIT3* is indeed a human RT disease gene, being discovered as a cause of renal agenesis and hypodysplasia^68^. Additionally, *Slit3* knockout mice have been reported to demonstrate renal agenesis, although this phenotype was only present in 20% of the animals analysed^69^.

Within the 22q11.2 deletion region, we have identified *Crkl* as a candidate kidney development gene. Notably, *Crkl* protein altering variants have been found in DiGeorge syndrome patients with congenital urinary abnormalities^40^, providing strong support for our classification of this gene as a RT development gene. *CRKL* has also been found as one of the keys genes for the normal development of both upper and lower genitourinary (GU) tracts, and its deletion at 22q11.2 is shown to cause urogenital birth defects^70^. Another gene in the 22q11.2 critical region predicted to be associated with RT development, *KLF8*, has been shown to be over expressed in renal cell carcinoma tissue as compared to non-tumour adjacent tissue^71^. siRNA knockdown of *KLF8* limited cellular growth and invasion capacity of human renal carcinoma cells in vitro. Therefore, *KLF8* likely plays a role in proliferation of renal carcinoma cells. Further investigation is needed to determine if *KLF8* also plays a role in developmental renal cell proliferation.

Overall, our classifier has identified several predicted RTM genes within our test datasets that have links to kidney or RT development. It is important to note that our classifier, whilst achieving superior accuracy to random guessing, still remains a computational tool which cannot be expected to achieve perfection for every gene status prediction. Looking forward, we propose that experimental analysis of the genes with highly confident RTM predictions will confirm or refute the role of these specific genes in RT development. Exploration of RTM patient exome and/or genome sequence datasets will reveal if these genes harbour deleterious variants in individuals with RTMs. Modelling deleterious variants in cell and animal models will enable deeper understanding of the developmental processes that these variants disrupt. Furthermore, the RTM gene predictions can be of use in determining which genes within an identified RTM genomic critical region or copy number variable region should be considered the most likely genetic candidates for causing disease. Our predictions may be informative for the analysis of sequence variation from RTM patients, to allow prioritisation of variants within genes of currently unknown RTM association status. Combining animal model analysis and RTM patient genome sequence analysis will provide strong evidence that genes with high confidence predictions are indeed linked to human RTMs, expanding our knowledge of the genetic causes of congenital kidney and lower urinary tract disease and expediting genetic diagnosis for RTM patients.

## Methods

### Data retrieval

We used the MGI database to compile a dataset comprising all mouse genes. Mouse genes were labelled as either RTM or non-RTM using the mutant mouse phenotype information from the IMPC and MGI databases (accessed on 15 October 2016). Only null alleles of mouse genes with known phenotypes resulting from single gene knockout (targeted deletions) experiments were included in this study. We defined the phenotype of a knockout mouse as RTM if the gene was known to be involved in renal development. These genes can potentially cause congenital renal developmental defects when mutated. A total of 10 phenotype terms in the MGI were used to classify a single gene knockout phenotype as RTM. These were: abnormal kidney morphology (MP:0002135), abnormal ureter morphology (MP:0000534), abnormal ureteropelvic junction morphology (MP:0011487), abnormal ureterovesical junction morphology (MP:0011488), abnormal urethra morphology (MP:0000537), abnormal urinary bladder morphology (MP:0000538), abnormal urinary system development (MP:0003942), abnormal urothelium morphology (MP:0003630), persistent cloaca (MP:0003129) and vesicoureteral reflux (MP:0001948). The RTM genes were also checked manually to find out which RT abnormalities are associated with them in the mouse. RTM genes were further verified by manually checking whether they are also critical to RT anomalies in humans. Genes with insufficient evidence of an associated RT phenotype in the mouse have been excluded. Additionally, renal ciliopathy genes have also been excluded from our RTM gene dataset. Mouse knockouts with phenotypes unrelated to any of these renal annotations were marked as non-RTM. Our datasets were restricted to protein-coding genes only. We further retrieved the Ensembl^72^ gene identifier and UniGene^73,74^ expression cluster identifier mapping to each MGI gene symbol. Encoded proteins for each mouse gene were determined from the UniProt database. Only the longest length protein isoform was analysed for each gene.

### Feature collection

We collected a number of gene and protein-sequence-based features to differentiate RTM and non-RTM phenotypes. Features including ‘gene length’, ‘% of GC content’, ‘transcript count’, ‘exon count’, ‘exon length’ and ‘intron length’ were computed based on the data retrieved from the Ensembl release 103 database of *Mus musculus* genes, using the Ensembl BioMart^75^ data mining tool. Gene expression data as transcripts per million (TPM) were obtained from the UniGene database for 13 embryonic developmental stages. The RNA-seq gene expression data were downloaded from the BGEE^76^ database which included 6 tissue types (11 weeks testis, 8 weeks fibroblast, 8 weeks heart, post-juvenile adult RTM, post-juvenile testis and 2 months skin). The Pepstats^77^ program was used to calculate protein length, molecular weight and amino acid composition. UniProt and WoLF PSORT^78^ program were used for subcellular localisation features. Other gene and protein-sequence-based features including evolutionary age, signal peptides, transmembrane domain, subcellular locations were obtained from Ensembl, SignalP^79^ and UniProt. Mouse protein–protein interaction (PPI) data were downloaded from the I2D^80^ v2.3 database, which is a database of known and predicted protein interactions for human, mouse, rat, fly, yeast and worm genomes. The ‘network analyser’ plugin of Cytoscape^81^ v3.1.1 and the Hub object Analyser (Hubba)^82^ web-based service were used to compute PPI network properties. GO terms were obtained using the ‘Functional Annotation’ tool of the web-based application DAVID^83^ v6.8. A detailed description of these features has been explained in previous studies^14,24^. Data on the chromosome location of mouse genes were obtained from Ensembl.

### Machine learning classifiers

A Random Forest classifier was developed using the publicly available Java based machine learning software Weka (version 3.8.2). The classifier was trained using the tenfold cross-validation method on a training dataset of RTM and non-RTM mouse genes, where the training dataset was randomly split into 10 equal datasets with 9 datasets being used for classifier training and the remaining part being used for testing. Training datasets with equal number of RTM and non-RTM genes were used to avoid bias towards the larger gene group. However, we could not find data for numerous features for a number of genes in the training datasets. These include: 10 features of the PPI network generated from known PPIs and gene expression across 13 developmental stages. Adjustments were made to these features by replacing their missing values with the respective feature mean values. Separate test datasets were also created from genes that have not been included in classifier training. Calculating the proportion of correctly predicted genes in the test datasets validated the performance of the classifier. The classifier generates a probability score to indicate the confidence level of a prediction outcome. This probability score is calculated by taking the average of all predictions made by the decision trees in the Random Forest. A score of 1 indicates that all trees agree to the same class prediction.

### Oversampling technique

Since our RTM and non-RTM datasets varied in the number of genes, we generated balanced training datasets containing an equal number of RTM and non-RTM mouse genes. The data imbalance was overcome by subsampling the non-RTM dataset at random^84^ and by generating synthetic instances of the RTM class using SMOTE. SMOTE is one of the most widely used oversampling techniques to solve class imbalances by generating synthetic samples for the minority class based upon the existing minority class samples. Each training dataset contained different subsets of RTM and non-RTM genes as a result of random selection.

### Feature selection

Accurate and reliable classification mainly relies upon the quality of the input features used to build the classifier; not all the features in the training dataset are useful. Usage of relevant features can reduce overfitting, optimise classification performance and decrease the training time. Feature selection was performed using the Information Gain method implemented in Weka, which estimates the rank of a feature by evaluating its information gain in the context of the classification target and selects only the most informative features for classification in order of significance^85^. The higher the value of the information gain is, the more important the feature is in determining the classification target.

### Performance measures

Performance of the predictive classifier was evaluated by several metrics which include accuracy, confusion matrix, precision and recall. Our classifier scores a prediction as TP (number of RTM genes correctly identified) or FP (number non-RTM genes incorrectly identified), or TN (number of non-RTM genes correctly identified) or FN (number of RTM genes incorrectly identified). Four metrics were estimated from these counts to assess how fit our classifier is in gene prediction: accuracy (proportion of true results); true positive rate (recall or sensitivity)–TPR; false positive rate–FPR; and precision, defined by the following equations: 

1 $$Accuracy=\frac{TP+TN}{TP+TN+FP+FN}$$

2 $$TPR= \frac{TP}{TP+FN} $$

3 $$FPR= \frac{FP}{FP+TN} (3)$$

4 $$Precision= \frac{TP}{TP+FP} (4)$$

Classifier performance was further evaluated from the area values of receiver operating curve (ROC) and precision-recall curve (PRC). The ROC area measures how well a classifier is performing in general, whereas the PRC area measures how well the classifier fits in identifying the samples from individual group. An area value of 1 represents an accurate prediction; a value of 0.5 represents a random guess.

### Statistical analysis

The statistical significance of each feature was determined using the non-parametric Mann–Whitney U test. We also used the Chi-squared (χ^2^) test to examine whether the frequencies of a feature in RTM and non-RTM dataset differ from each other. All statistical tests were performed using the statistics software package SPSS v23. Data visualisation was performed using R^86^.

### Supplementary Information

Supplementary Tables.

## Data Availability

All data generated or analysed during this study are included in this article (and its Supplementary Information files).

## References

[1] Neild GH (2010). Primary renal disease in young adults with renal failure. Nephrol. Dial. Transplant..

[2] Plumb L (2018). demography of the UK paediatric renal replacement therapy population in 2016. Nephron.

[3] Westland R, Renkema KY, Knoers NV (2021). Clinical integration of genome diagnostics for congenital anomalies of the kidney and urinary tract. Clin. J. Am. Soc. Nephrol..

[4] Woolf AS, Lopes FM, Ranjzad P, Roberts NA (2019). Congenital disorders of the human urinary tract: Recent insights from genetic and molecular studies. Front. Pediatr..

[5] Adalat S (2009). HNF1B mutations associate with hypomagnesemia and renal magnesium wasting. J. Am. Soc. Nephrol..

[6] Weber S (2006). Prevalence of mutations in renal developmental genes in children with renal hypodysplasia: Results of the ESCAPE study. J. Am. Soc. Nephrol..

[7] Groen I’nt Woud S (2016). Maternal risk factors involved in specific congenital anomalies of the kidney and urinary tract: A case–control study. Birth Defects Res. Part A Clin. Mol. Teratol..

[8] Woolf AS (2011). Environmental influences on renal tract development: A focus on maternal diet and the glucocorticoid hypothesis. Klin. Padiatr..

[9] Baştanlar, Y. & Özuysal, M. Introduction to machine learning. *miRNomics: MicroRNA biology and computational analysis*, 105–128 (2014). 10.1007/978-1-62703-748-8_7 24272434

[10] Libbrecht MW, Noble WS (2015). Machine learning applications in genetics and genomics. Nat. Rev. Genet..

[11] Bakheet TM, Doig AJ (2009). Properties and identification of human protein drug targets. Bioinformatics.

[12] Bakheet TM, Doig AJ (2010). Properties and identification of antibiotic drug targets. BMC Bioinform..

[13] Bull SC, Doig AJ (2015). Properties of protein drug target classes. PLoS ONE.

[14] Tian D (2018). Identifying mouse developmental essential genes using machine learning. Dis. Models Mech..

[15] Yang W (2014). Identification of genes and pathways involved in kidney renal clear cell carcinoma. BMC Bioinform..

[16] Guan, Y., Martini, S. & Mariani, L. H. in *Seminars in Nephrology.* 237–244 (Elsevier). 10.1016/j.semnephrol.2015.04.003 PMC4518206 26215861

[17] López-Bigas N, Ouzounis CA (2004). Genome-wide identification of genes likely to be involved in human genetic disease. Nucleic Acids Res..

[18] Oliver PL, Bitoun E, Davies KE (2007). Comparative genetic analysis: The utility of mouse genetic systems for studying human monogenic disease. Mamm. Genome.

[19] Rangarajan A, Weinberg RA (2003). Comparative biology of mouse versus human cells: Modelling human cancer in mice. Nat. Rev. Cancer.

[20] Bult CJ (2008). The mouse genome database (MGD): mouse biology and model systems. Nucleic Acids Res..

[21] Koscielny G (2014). The international mouse phenotyping consortium web portal, a unified point of access for knockout mice and related phenotyping data. Nucleic Acids Res..

[22] Hamosh A, Scott AF, Amberger JS, Bocchini CA, McKusick VA (2005). Online Mendelian Inheritance in Man (OMIM), a knowledgebase of human genes and genetic disorders. Nucleic Acids Res..

[23] Murugapoopathy V, Gupta IR (2020). A primer on congenital anomalies of the kidneys and urinary tracts (CAKUT). Clin. J. Am. Soc. Nephrol..

[24] Kabir M, Barradas A, Tzotzos GT, Hentges KE, Doig AJ (2017). Properties of genes essential for mouse development. PLoS ONE.

[25] Apweiler R (2004). UniProt: The universal protein knowledgebase. Nucleic Acids Res..

[26] Consortium, U. UniProt: A hub for protein information. *Nucleic acids research* **43**, D204-D212 (2015). 10.1093/nar/gku989 PMC4384041 25348405

[27] Ashburner M (2000). Gene ontology: Tool for the unification of biology. Nat. Genet..

[28] Stark C (2006). BioGRID: A general repository for interaction datasets. Nucleic Acids Res..

[29] Bader GD, Betel D, Hogue CW (2003). BIND: The biomolecular interaction network database. Nucleic Acids Res..

[30] Chen C (2009). Mouse Piwi interactome identifies binding mechanism of Tdrkh Tudor domain to arginine methylated Miwi. Proc. Natl. Acad. Sci..

[31] Hermjakob H (2004). IntAct: An open source molecular interaction database. Nucleic Acids Res..

[32] Lynn DJ (2008). InnateDB: facilitating systems-level analyses of the mammalian innate immune response. Mol. Syst. Biol..

[33] Xenarios I (2000). DIP: The database of interacting proteins. Nucleic Acids Res..

[34] Zanzoni A (2002). MINT: A Molecular INTeraction database. FEBS Lett..

[35] Wang J (2006). A protein interaction network for pluripotency of embryonic stem cells. Nature.

[36] He H, Garcia EA (2009). Learning from imbalanced data. IEEE Trans. Knowl. Data Eng..

[37] Visa, S. & Ralescu, A. in *Proceedings of the Sixteen Midwest Artificial Intelligence and Cognitive Science Conference.* 67–73 (sn).

[38] Chawla NV, Bowyer KW, Hall LO, Kegelmeyer WP (2002). SMOTE: Synthetic minority over-sampling technique. J. Artif. Intell. Res..

[39] Kobrynski LJ, Sullivan KE (2007). Velocardiofacial syndrome, DiGeorge syndrome: the chromosome 22q11. 2 deletion syndromes. Lancet.

[40] Lopez-Rivera E (2017). Genetic drivers of kidney defects in the DiGeorge syndrome. N. Engl. J. Med..

[41] Darlow JM (2017). Genome-wide linkage and association study implicates the 10q26 region as a major genetic contributor to primary nonsyndromic vesicoureteric reflux. Sci. Rep..

[42] Motenko H, Neuhauser SB, O’keefe M, Richardson JE (2015). MouseMine: a new data warehouse for MGI. Mamm. Genome.

[43] Breiman L (2001). Random forests. Mach. Learn..

[44] Hall M (2009). The WEKA data mining software: An update. ACM SIGKDD Explor. Newsl..

[45] Acencio ML, Lemke N (2009). Towards the prediction of essential genes by integration of network topology, cellular localization and biological process information. BMC Bioinform..

[46] Bureau A (2005). Identifying SNPs predictive of phenotype using random forests. Genet. Epidemiol. Off. Publ. Int. Genet. Epidemiol. Soc..

[47] Yuan Y, Xu Y, Xu J, Ball RL, Liang H (2012). Predicting the lethal phenotype of the knockout mouse by integrating comprehensive genomic data. Bioinformatics.

[48] Kohavi, R. in *Ijcai.* 1137–1145 (Montreal, Canada).

[49] Breiman L, Friedman JH, Olshen RA, Stone CJ (1984). Classification and Regression Trees.

[50] Chen, T. & Guestrin, C. in *Proceedings of the 22nd acm sigkdd International Conference on Knowledge Discovery and Data Mining.* 785–794.

[51] Chen, T. *et al.* Xgboost: Extreme gradient boosting. *R package version 0.4-2* **1**, 1-4 (2015).

[52] Cortes C, Vapnik V (1995). Support-vector networks. Mach. Learn..

[53] Fuchs H (2016). The first Scube3 mutant mouse line with pleiotropic phenotypic alterations. G3 Genes Genomes Genet..

[54] Cai A (2022). Genetic inactivation of Semaphorin 3C protects mice from acute kidney injury. Kidney Int..

[55] Reidy K, Tufro A (2011). Semaphorins in kidney development and disease: Modulators of ureteric bud branching, vascular morphogenesis, and podocyte-endothelial crosstalk. Pediatr. Nephrol..

[56] Combes AN (2019). Single cell analysis of the developing mouse kidney provides deeper insight into marker gene expression and ligand-receptor crosstalk. Development.

[57] Vidal VP (2020). R-spondin signalling is essential for the maintenance and differentiation of mouse nephron progenitors. Elife.

[58] Haworth K (2007). Expression of the Scube3 epidermal growth factor-related gene during early embryonic development in the mouse. Gene Expr. Patterns.

[59] Lin Y-C (2021). SCUBE3 loss-of-function causes a recognizable recessive developmental disorder due to defective bone morphogenetic protein signaling. Am. J. Human Genet..

[60] Weber S (2008). SIX2 and BMP4 mutations associate with anomalous kidney development. J. Am. Soc. Nephrol..

[61] Morris MR (2011). Genome-wide methylation analysis identifies epigenetically inactivated candidate tumour suppressor genes in renal cell carcinoma. Oncogene.

[62] Khouja HI (2022). Multi-staged gene expression profiling reveals potential genes and the critical pathways in kidney cancer. Sci. Rep..

[63] Potter AS, Drake K, Brunskill EW, Potter SS (2019). A bigenic mouse model of FSGS reveals perturbed pathways in podocytes, mesangial cells and endothelial cells. PLoS ONE.

[64] Villegas G, Tufro A (2002). Ontogeny of semaphorins 3A and 3F and their receptors neuropilins 1 and 2 in the kidney. Mech. Dev..

[65] Maschietto M (2011). Temporal blastemal cell gene expression analysis in the kidney reveals new Wnt and related signaling pathway genes to be essential for Wilms' tumor onset. Cell Death Dis..

[66] Natrajan R (2006). Array CGH profiling of favourable histology Wilms tumours reveals novel gains and losses associated with relapse. J. Pathol. A J. Pathol. Soc. Great Br. Irel..

[67] Dwivedi, N., Jamadar, A., Mathew, S., Fields, T. A. & Rao, R. Myofibroblast depletion reduces kidney cyst growth and fibrosis in autosomal dominant polycystic kidney disease. *Kidney Int.* (2022). 10.1016/j.kint.2022.08.036 PMC9822873 36273656

[68] Sanna-Cherchi S (2017). Exome-wide association study identifies GREB1L mutations in congenital kidney malformations. Am. J. Human Genet..

[69] Liu J (2003). Congenital diaphragmatic hernia, kidney agenesis and cardiac defects associated with Slit3-deficiency in mice. Mech. Dev..

[70] Haller M, Mo Q, Imamoto A, Lamb DJ (2017). Murine model indicates 22q11.2 signaling adaptor CRKL is a dosage-sensitive regulator of genitourinary development. Proc. Natl. Acad. Sci..

[71] Fu W-J (2010). Small interference RNA targeting Krüppel-like factor 8 inhibits the renal carcinoma 786–0 cells growth in vitro and in vivo. J. Cancer Res. Clin. Oncol..

[72] Hubbard T (2002). The Ensembl genome database project. Nucleic Acids Res..

[73] Pontius, J. U., Wagner, L. & Schuler, G. D. 21. UniGene: A unified view of the transcriptome. *The NCBI Handbook. Bethesda, MD: National Library of Medicine (US), NCBI* (2003).

[74] Stanton J-AL, Macgregor AB, Green DP (2003). Identifying tissue-enriched gene expression in mouse tissues using the NIH UniGene database. Appl. Bioinform..

[75] Smedley D (2009). BioMart–biological queries made easy. BMC Genom..

[76] Bastian, F. *et al.* in *International Workshop on Data Integration in the Life Sciences.* 124–131 (Springer).

[77] Rice P, Longden I, Bleasby A (2000). EMBOSS: The European molecular biology open software suite. Trends Genet..

[78] Horton P (2007). WoLF PSORT: Protein localization predictor. Nucleic Acids Res..

[79] Petersen TN, Brunak S, Von Heijne G, Nielsen H (2011). SignalP 4.0: Discriminating signal peptides from transmembrane regions. Nat. Methods.

[80] Brown KR, Jurisica I (2007). Unequal evolutionary conservation of human protein interactions in interologous networks. Genome Biol..

[81] Shannon P (2003). Cytoscape: a software environment for integrated models of biomolecular interaction networks. Genome Res..

[82] Lin C-Y (2008). Hubba: Hub objects analyser–a framework of interactome hubs identification for network biology. Nucleic Acids Res..

[83] Huang DW (2007). DAVID Bioinformatics Resources: Expanded annotation database and novel algorithms to better extract biology from large gene lists. Nucleic Acids Res..

[84] Vitter JS (1985). Random sampling with a reservoir. ACM Transact. Math. Softw. (TOMS).

[85] Han, J., Kamber, M. & Pei, J. Data mining: Concepts and techniques. (Elsevier, 2011).

[86] Team, R. C. R. A language and environment for statistical computing. (2013).

